# Coexistence of coyotes (*Canis latrans*) and red foxes (*Vulpes vulpes*) in an urban landscape

**DOI:** 10.1371/journal.pone.0190971

**Published:** 2018-01-24

**Authors:** Marcus A. Mueller, David Drake, Maximilian L. Allen

**Affiliations:** Department of Forest and Wildlife Ecology, University of Wisconsin, Madison, Wisconsin, United States of America; Sichuan University, CHINA

## Abstract

Urban environments are increasing worldwide and are inherently different than their rural counterparts, with a variety of effects on wildlife due to human presence, increased habitat fragmentation, movement barriers, and access to anthropogenic food sources. Effective management of urban wildlife requires an understanding of how urbanization affects their behavior and ecology. The spatial activity and interactions of urban wildlife, however, have not been as rigorously researched as in rural areas. From January 2015 to December 2016, we captured, radio-collared, and tracked 11 coyotes and 12 red foxes in Madison, WI. Within our study area, coyotes strongly selected home ranges with high proportions of natural areas; conversely, red foxes selected home ranges with open space and moderately developed areas. Use of highly developed areas best explained variation among individual home range sizes and inversely affected home range size for coyotes and red foxes. Coyote and red fox home ranges showed some degree of spatial and temporal overlap, but generally appeared partitioned by habitat type within our study area. Coyotes and red foxes were both active at similar times of the day, but their movement patterns differed based on species-specific habitat use. This spatial partitioning may promote positive co-existence between these sympatric canids in urban areas, and our findings of spatial activity and interactions will better inform wildlife managers working in urban areas.

## Introduction

Urbanization is an agent of change for the biotic environment and has strong effects on wildlife populations [1–2]. As of 2014, 88% of Americans lived in urban environments and urban land acreage has quadrupled since 1945 [3]. This trend is likely to continue, as over five billion people are projected to live in urban areas worldwide by 2030 [4], highlighting the need to understand the effects of urbanization on biotic communities. Urban environments are inherently different from their rural counterparts, primarily due to human presence, but also due to increased habitat fragmentation, movement barriers (i.e., roads and development), and access to anthropogenic sources of food as well as mortality [5–6]. Natural disturbances, such as fire, are suppressed and replaced with a host of anthropogenic disturbances—including construction, landscaping, and recreation—all with different effects on urban biological communities [7–8]. Urbanization affects biodiversity and has a variety of impacts on wildlife populations living near and within city limits, and is a primary reason for the local extirpation of many species [1, 9].

Many carnivores are sensitive to urbanization due to expansive home ranges, large body size, and high energetic demands [10–11]. Urbanization may change how certain carnivore species coexist on the landscape with other sympatric carnivores by altering the distribution and activity of apex carnivores and having direct and indirect effects on mesocarnivore communities [8]. The mesocarnivore release theory describes one of these potential effects whereby removing a top carnivore may cause populations of a subordinate to rapidly increase in its absence [10]. A behavioral effect is that carnivores in urban areas tend to shift their traveling and hunting activity to nighttime, when human activity decreases [12–13]. Carnivore richness in response to urbanization is species-dependent, but is generally lower as urbanization intensifies [14], and road density restricts movements while threatening carnivore population viability due to vehicle mortality and constrained gene flow [15]. However, research has shown that several carnivore species can successfully inhabit, and even thrive, in human-dominated landscapes [6, 16]. Relative to non-urban conspecifics, many urban carnivores exhibit smaller home ranges and sometimes even higher population densities due to the exploitation of stable, anthropogenically altered sources of food and shelter [6, 17].

Throughout North America, coyotes (*Canis latrans*) and red foxes (*Vulpes vulpes*) are two adaptive canids capable of exploiting the urban landscape [6]. Over the last century, coyotes have expanded their range across the continental United States and are present in many North American cities [18–19]. This range expansion is due to their flexibility in satisfying habitat needs and adaptability to a changing landscape, as well as the wide-scale extirpation of grey wolves (*Canis lupus*), a larger competitor known to limit sympatric coyote populations [20]. In Europe, red foxes were officially recorded in cities as early as the 1930’s but may have been present much earlier [21–22]. It is unclear when red foxes colonized North American cities, but literature suggests they have been present in Midwestern urban areas since the 1960’s [22–23]. Competing hypotheses exist about what caused red fox expansion into urban environments, ranging from refuge from disease outbreak [24] to competitive exclusion by coyotes [25–26].

Coyotes and red foxes are often competitors that use similar resources and may have top-down effects on prey communities [25, 27–28]. In rural contexts, red foxes and coyotes often spatiotemporally partition habitat resources [25, 29]. This may also take the form of interference competition, where sympatric groups of coyotes and red foxes display interspecific territoriality as a mechanism to regulate populations [27–28]. Coyotes act as apex carnivores in many urban areas [8, 30], and both species can contribute to ecosystem structure through top-down regulation of prey species [10, 26, 31]. Coyotes and red foxes occur and overlap in many urban areas, but in contrast to rural areas the nature of their coexistence and competition has not been rigorously studied [26].

A potentially confounding factor in coyote-fox interactions is that human activities drive the spatial patterns, ecological processes, and dynamics of urban systems [32–34]. Because humans have the potential to directly and indirectly affect the spatiotemporal presence of urban coyotes and red foxes, it is particularly important to understand the spatial ecology of these species relative to human development and activity. Although coyotes thrive in many human-dominated landscapes, they usually do so by spending most of their time in urban areas with low human activity [35]. In rural settings, red foxes avoid coyotes in both space and time [25, 28], but are not generally restricted by human activity and have been documented to exist in close proximity to humans in urban areas [36].

Our objective was to determine if and how the sympatric coexistence occurred between coyotes and red foxes in a human-dominated landscape. We hypothesized that urban coyotes would primarily use natural areas or areas with low human development, while red foxes would use areas with moderate and high human development. However, due to the similar nature of coyote and red fox resource requirements and abundance of anthropogenically altered food and shelter resources, we expected to see spatial overlap between species. Specifically, our research aimed to answer: 1) What factors drive home range size for urban coyotes and red foxes? 2) Do coyotes and red foxes coexist in urban areas by selecting for different urban habitats relative to land use? 3) Does coyote and red fox movement differ within the urban landscape, especially relative to human development or interspecific canid presence?

## Methods

### Study area

**Fig 1:**
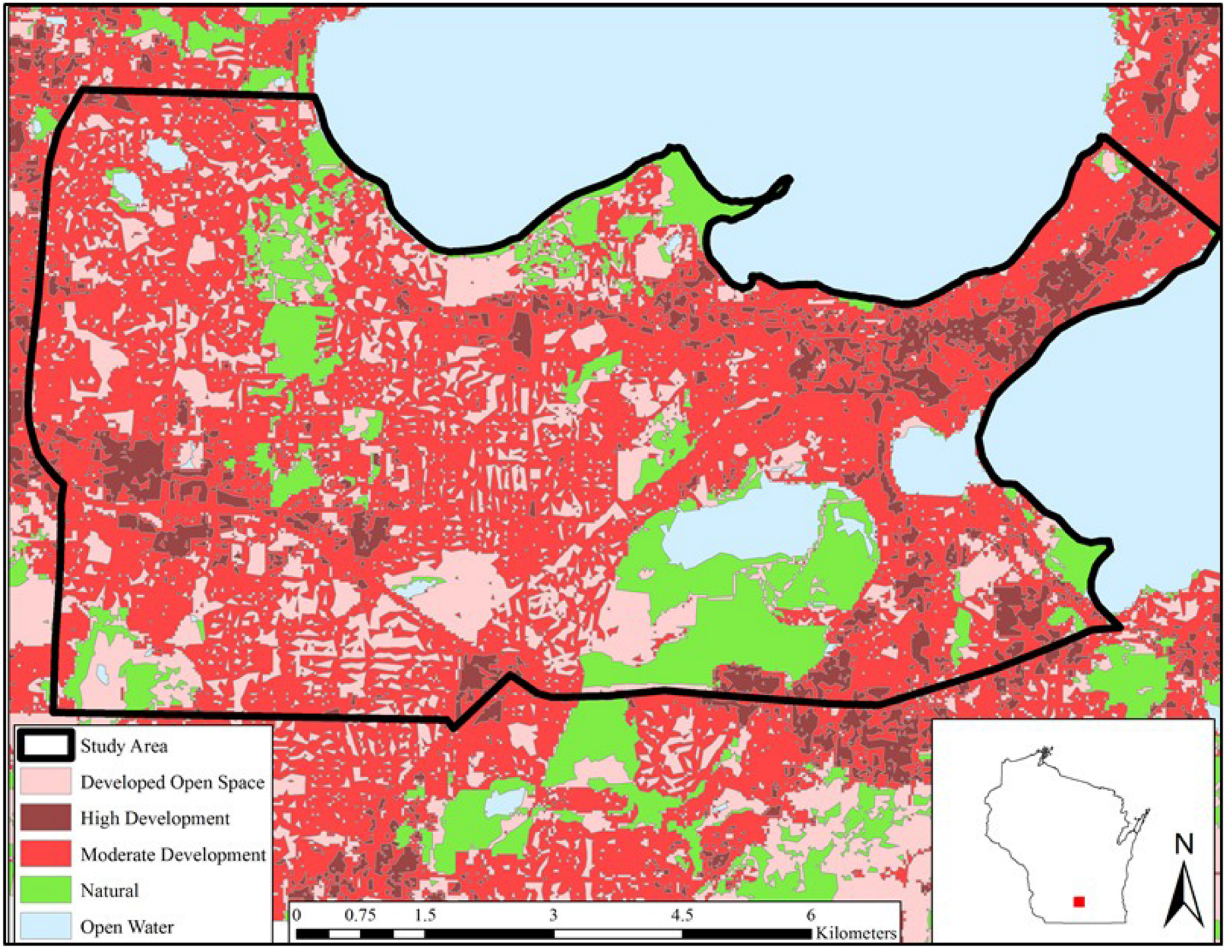
Land cover classifications within our study area. Madison, WI, 2015–2016.

Our study area was located in Madison, WI (Fig 1). Madison is the second largest city in Wisconsin, with a population of 245,000 people [3]. Mean temperatures are -10.4°^C^ in winter to 20.6°^C^ in summer with mean yearly precipitation of 87.38 cm [37]). Our 7108.9ha study area was comprised of 11.85% (842.43ha) of natural areas, and88.15% (6266.57 ha) of non-natural area. Across the study area, there were on average 10.04km of roads per km2. Our study area encompassed the University of Wisconsin-Madison (UW) campus, along with a mosaic of residential, commercial, and semi-isolated natural areas bounded by developed roads and neighborhoods. Our study area also encompassed several public natural areas, including the UW Lakeshore Nature Preserve (121 ha), Owen Conservation Park (39 ha), and UW Arboretum (486 ha). Bordering the southwestern shore of Lake Mendota, the UW Lakeshore Nature Preserve consisted of upland broadleaf deciduous forests (oak (*Quercus spp*.), hickory (*Carya spp*.), ash (*Fraxiunus spp*.), basswood (*Tilia americana*)), a restored tallgrass prairie (indiangrass (*Sorghastrum nutans*), big bluestem (*Andropogon gerardi*), little bluestem (*Schizachyrium scoparium*)), and several small wetlands (cattail (*Typha spp*.)). Owen Conservation Park was part of the Madison Parks system and was composed of both restored tallgrass prairies and oak savannas. The UW Arboretum consisted of many major habitat types, including upland broadleaf deciduous forests, restored tallgrass prairies and oak savannas, human-planted coniferous forests, and various wetland complexes. Wooded corridors existed throughout our study area.

### Capture and monitoring

We live-captured adult coyotes and red foxes from January to April 2015 and November 2015 to April 2016 using cable restraints. This study was conducted in strict accordance with followed all ethical capture procedures [38]. Our animal handling protocol were approved by the University of Wisconsin Institutional Animal Care and Use Committee (Protocol A01559), and Wisconsin Department of Natural Resources (Permit # SCP-SOD-001-2014). We made extensive efforts to minimize or eliminate suffering throughout our handling process, and no animals were killed during our study. We chemically immobilized each trapped canid intramuscularly (IM) using ketamine (10 mg/kg for coyotes, 4 mg/kg for red foxes) and xylazine (2mg/kg). We weighed each animal, assessed physical condition (i.e., eyes, teeth, coat, body condition), and collected other biological samples. Finally, we fitted each animal with ear tags and a very high frequency (VHF) radio collar (Advanced Telemetry Systems, Isanti, MN; Model # M1950 for red fox and M2220B for coyote) or satellite Global Positioning System (GPS) collar (Lotek Wireless Fish & Wildlife Monitoring, Newmarket, ON; Model #G5C175C). We administered Yohimbine (0.1 mg/kg) to reverse immobilization for all canids.

We attempted to locate each VHF-collared animal via radio receiver at least once per week for the entire duration that the radio collar functioned and remained on the animal, or the animal remained alive. We triangulated each location based on the intersections of ≥3 telemetry bearings taken within a maximum of 15 minutes of each other to reduce error based on animal movement [39]. We also located animals using GPS readings when we visually observed individuals, but these observations were rare and did not appear to affect the animal’s natural behavior. To ensure the accuracy of triangulations, we plotted the telemetry bearings and estimated the location of the animal on a laptop computer to proof locations in the field (Radio-Tracker, John Cary, University of Wisconsin, Madison, WI). During the weekly location of each animal, we tracked them for a 5-hour period, where we located the focal animal once per hour during that period. We systematically rotated weekly tracking periods around the 24-hour clock to ensure that we documented temporal variation in activity. We programmed GPS collars to collect locations at hourly intervals.

### Data variables

We used ArcGIS 10.4 (Redlands, CA: Environmental Systems Research Institute) to delineate habitat types relative to human development in our study area based on National Land Cover Data (NLCD) [40]. We grouped NLCD habitat types into five categories based on previous urban canid research [25]: Developed open space (OPEN; i.e., turf fields, non-forested parks, and cemeteries); moderate intensity development (MDEV; i.e., 20–79% impervious surface, residential neighborhoods); high intensity development (HDEV; i.e., ≥80% impervious surface, industrial and commercial land); non-developed (NATR; i.e., natural areas, including forest, grassland, emergent wetlands); and water (WATR; i.e., open bodies of water) (Fig 1). We obtained local population density (humans/km^2^) data from the National Historical Geographic Information System [41], based geographically on the most recent U.S.A. Census blocks.

We calculated time of day based on local sunrise and sunset times using 4 periods: sunrise (time of sunrise ± 2 hours), sunset (time of sunset ± 2 hours), day (began after sunrise period and lasted until sunset period), and night (began after sunset period and lasted until sunrise period). This approach led to uneven temporal lengths of time periods, but more accurately reflected seasonal changes to photoperiods.

We examined radio location data annually and by seasons based on biologically meaningful periods of life for each species: breeding (red fox = November to February, coyote = December to March), pup-rearing (red fox = March to June, coyote = April to July), and non-breeding (red fox = July to October, coyote = August to November) [19, 42].

### Statistical analysis

We estimated home ranges using minimum convex polygons (MCP) [43] for individual coyotes and red foxes using the *adehabitatHR* package in R (version 2.11.1; R Foundation for Statistical Computing, Vienna, Austria). To limit autocorrelation, we used a subset of every other telemetry point (i.e., the first, third, and fifth telemetry location, when available) from each 5-hour tracking period. We then calculated 95% MCPs to estimate the individual’s home range, and 50% MCPs to estimate core use areas annually. We also calculated seasonal home ranges for each of the three seasons for individuals, where we included canids with ≥15 independent telemetry locations within the respective season. While this allowed us to include the greatest number of canids in our seasonal analysis, it should be noted that this sample size is below most thresholds for minimum sample sizes for MCP home range estimation [44].

We used the *adehabitatHS* package [45] in R to perform multi-scaled compositional analyses for each individual canid. To avoid the issues of autocorrelation associated with other methods of analyzing telemetry data, this method treats individual animals as sampling units, instead of individual locations. This technique presents habitat use as a value ranging from 0 to 1, where the sum of all values equals 1. The resulting values represent selection (use relative to availability), and to what degree each category was selected [46]. Values greater that 1 represent habitat types selected for, values less than 1 represent habitat types selected against, and values of 1 represent no selection. We analyzed habitat selection on two scales: 2^nd^ order (home range) considered broad selection of an individual’s home range compared to available habitat, and 3^rd^ order (location) considered the use of actual locations compared to available habitat within an individual’s home range [46]. Because HDEV was absent from 36% of coyote home ranges, we chose to omit it from 3^rd^ order analysis to avoid having to censor more individuals from this analysis and further reduce our sample size.

We used Pianka’s index to quantify overlap in habitat use by coyotes and red foxes during different seasons [25, 47]. This index compared the percent use of habitat categories and resulted in a value ranging from 0 to 1 (0 suggested no overlap in habitat use and 1 suggested total overlap). We calculated this index on two different scales: the home range level indexed the overlap of the composition of coyote and red fox home ranges, and the location level indexed the overlap of the use of habitats available within home ranges.

We analyzed the potential drivers of home range size within our study area using an AIC modeling framework using 12 *a-priori* models (Tables 1 and 2). We analyzed annual and seasonal home ranges separately. We fit a linear model (annual) and linear mixed-effects model (seasonal) using 95% MCP size as our dependent variable. When no single top model was evident, we used model averaging across our top candidates (ƩAIC_w_ > 0.90) [48].

**Table 1:** Explanatory variables for modeling changes in annual and seasonal home range size for urban coyotes and red foxes in Madison, WI, 2015–2016.

Variable	Abbreviation	Description
Species	SPEC	Species of Animal (Coyote/Red Fox)
Sex	SEX	Sex of animal (M/F)
High Development	HDEV	Percent of home range made up by High Development (Commercial, Industrial)
Moderate Development	MDEV	Percent of home range made up by Moderate Development (Residential)
Developed Open	OPEN	Percent of home range made up by Developed Open (Lawns, Parks, turf fields)
Natural	NATR	Percent of home range made up by Natural (Green space)
Season	SEAS	Biological season of home range (Breeding, Non-breeding, Pup-rearing)

**Table 2:** Potential explanatory models to explain changes in home ranges sizes for urban canids in Madison, WI, 2015–2016.

Name	Variables	Description
Sex	SEX	Home range size will be driven by the sex of the animal because members of each sex have different energetic requirements and social roles. [19, 42]
Season	SEAS	Home range size will be driven by biological season because of the changes in energetic requirements and behaviors. [19, 42]
Species	SPEC	Home range size will be driven by species because species have different ecological roles and therefore use available habitat differently. [19, 42]
Species and Sex	SPEC * SEX	Home range size will be driven by the interaction between an individual animal’s species and sex because individual animals have different energetic demands and life history traits. [19, 42]
Species and Season	SPEC * SEAS	Home range size will be driven by the interaction between species and the time of year, because different species have different energetic requirements and life history traits depending on the time of year. [19, 42]
Species, Sex, Season	SPEC * SEX * SEAS	Home range size will be driven by the interaction between an individual animal’s species and sex because different animals have different energetic demands and life history traits at different times of the year. [19, 42]
Species-specific Use of NATR	SPEC * NATR	Species-specific use of natural habitat will drive home range size because natural areas provide traditionally suitable habitat for canids. [19, 22, 42, 49]
Species-specific Use of OPEN	SPEC * OPEN	Species-specific use of developed open habitat will drive home range size because areas such as cemeteries and parks may provide resources to support canids. [19, 22, 42, 49]
Species-specific use of MDEV	SPEC * MDEV	Species-specific use of moderately developed habitat will drive home range size because residential areas may provide resources to support canids. [19, 22, 42, 49]
Species-specific use of HDEV	SPEC * HDEV	Species-specific use of highly developed habitat will drive home range size because highly developed areas typically mean lower quality habitat. [19, 22, 42, 49]
Species-specific use of NATR and OPEN	SPEC * NATR + SPEC * OPEN	Species-specific use of natural and developed open habitat will drive home range size because these habitats may provide resources to support canids. [19, 22, 42, 49]
Species-specific use of NATR and MDEV	SPEC * NATR + SPEC * MDEV	Species-specific use of natural and moderately developed habitat will drive home range size because these habitats may provide resources to support canids. [19, 22, 42, 49]

We estimated canid movement activity by calculating the distance and direction between subsequent telemetry locations within a 5-hour tracking period (hereafter referred to as “steps”). To account for user-error influencing the time between locations (i.e., temporarily losing radio-contact with a canid), we excluded any steps that were calculated using locations greater than 72min (1.2hr) or less than 48min (0.8hr) apart. We classified the start of each step by species, sex, date, time of day, biological season (depending on species), and habitat type. In addition, we categorized red fox steps based on if they were within a coyote’s 50% MCP, outside of the 50% MCP but within the 95% MCP, or outside of the 95% MCP. We also examined if a red fox was moving in the direction of the nearest coyote 50% MCP by using the bearing from the origin of the step to the center of the 50% MCP +/- 90°. In each analysis, we used univariate analysis-of-variance (ANOVA) to investigate the influence of spatial and temporal variation on canid movement, followed by a post-hoc Tukey HSD test to identify habitat type and season.

## Results

### Overview

We captured and tracked 11 coyotes (n_male_ = 7, n_female_ = 4) and 12 red foxes (n_male_ = 8, n_female_ = 4) from January 2015 to December 2016. We excluded four foxes (n_male_ = 3, n_female_ = 1) from analyses because of a low number of fixes due to mortality or collar failure. We collected 5,729 total locations (n_coyote_ = 4382, ${\bar{x}}_{\text{coyote}}=365.2$, range_coyote_ = 36–2311; n_fox_ = 1347, ${\bar{x}}_{\text{fox}}=122.5$, range_fox_ = 2–458). Mean number of days on air were 282.3 (±60.7 SE) for coyote, and 243.6 (±71.6 SE) for red fox.

### Home ranges

**Fig 2:**
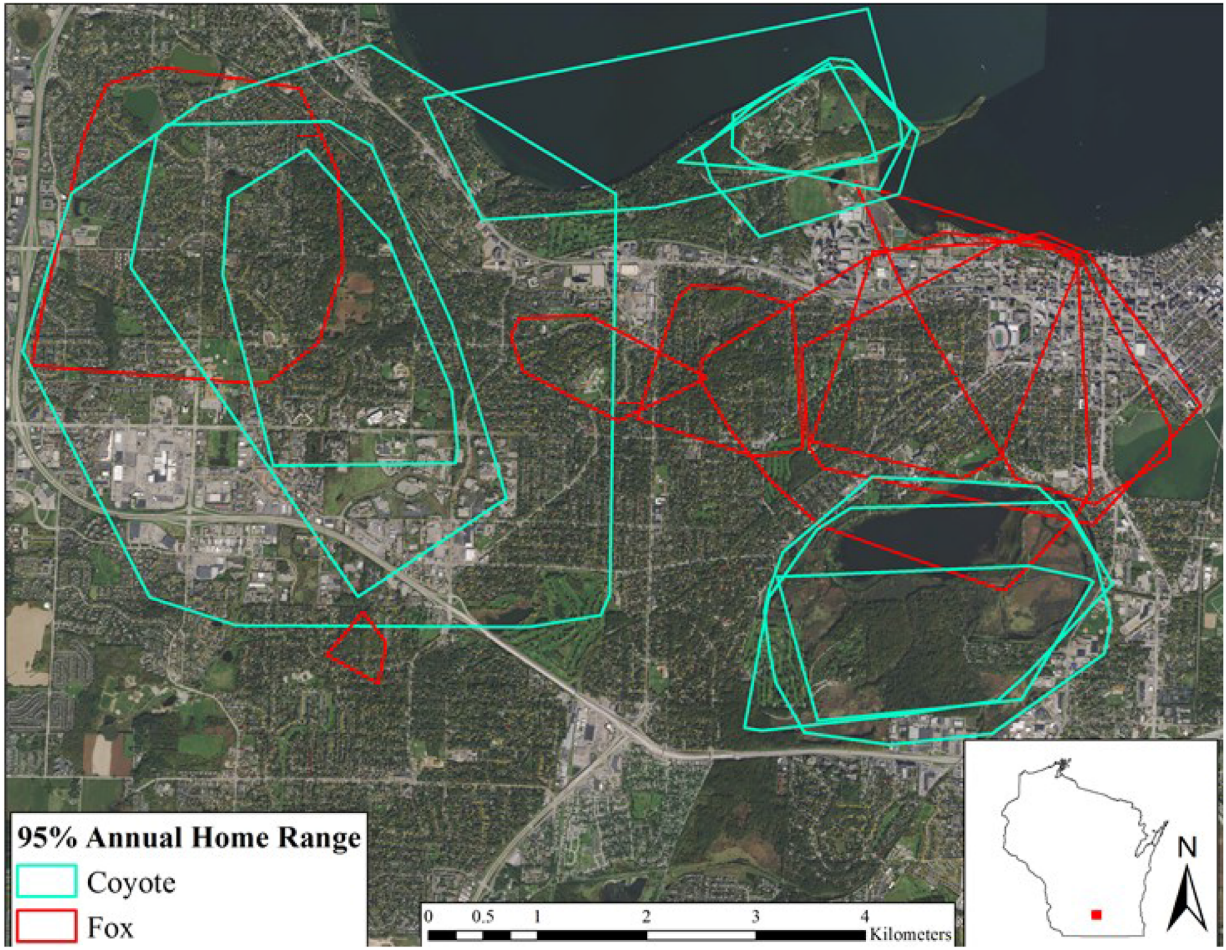
Annual 95% minimum convex polygon home ranges for coyotes and red foxes in Madison, WI, 2015–2016.

We calculated annual (Table 3, Fig 2) and seasonal (Table 4) 95% MCPs for each coyote (n = 11) and red fox (n = 8) from January 2015 to December 2016.

**Table 3:** Annual home range sizes (km^2^) for coyotes and red foxes in Madison, WI, 2015–2016.

	Coyote			Red Fox		
	All (M & F)	Male	Female	All (M & F)	Male	Female
Mean	5.79	7.46	2.89	3.99	5.63	2.34
Range	0.93–23.02	2.04–23.04	0.93–5.10	0.19–8.18	3.64–8.18	0.19–6.42
SE	0.87	0.94	1.06	2.18	1.06	1.40
n	11	7	4	8	4	4

**Table 4:** Seasonal home range sizes (km^2^) for coyotes and red foxes in Madison, WI, 2015–2016.

	Coyote			Red Fox		
	Breeding	Pup-rearing	Non-breeding	Breeding	Pup-rearing	Non-breeding
Mean	4.79	2.66	4.55	3.19	2.52	3.93
Range	0.79–16.18	0.49–4.98	0.79–13.11	0.16–6.63	0.40–4.89	0.86–6.22
SE	1.40	0.59	2.18	1.26	0.62	0.89
n	10	9	6	6	6	5

Breeding season refers to the 4 months prior to birth of pups, pup-rearing refers to the 4 months after birth, and non-breeding are the remaining 4 months of the year.

For annual home ranges, our top model, “Species-specific Use of HDEV,” (AIC_w_ = 0.97) was the only model with substantial support (Table 5). In our top model, species (SPEC) had a positive effect on home range size while HDEV was constant (ß_SPEC_ = 0.63). HDEV had a positive effect on home range size for coyotes (ß_HDEV_ = 2.22). The interaction between SPEC and HDEV had a negative effect on home range size (ß_SPEC:HDEV_ = -2.02).

**Table 5:** Results of *a-priori* model comparisons for selecting factors driving annual home range size in urban coyotes and red foxes in Madison, WI, 2015–2016.

Name	AICc	ΔAICc	AIC_w_	Cumulative AIC_w_
Species-specific use of HDEV	109.74	0.00	0.97	0.97
Sex	118.07	8.34	0.02	0.99
Species-specific use of NATR	120.14	10.40	0.01	1.00
Species	121.16	11.42	0.00	1.00
Species-specific use of MDEV	123.33	13.60	0.00	1.00
Species and Sex	124.62	14.89	0.00	1.00
Species-specific use of OPEN	126.69	16.95	0.00	1.00
Species-specific use of NATR and OPEN	128.19	18.46	0.00	1.00
Species-specific use of NATR and MDEV	129.24	19.50	0.00	1.00

For seasonal home ranges, our top models were “Species-specific use of NATR and OPEN” (AIC_w_ = 0.60), and “Species-specific use of NATR and MDEV” (AIC_w_ = 0.39) (Table 6). After model averaging, SPEC, or red fox home ranges compared to coyote (ß_SPEC_ = -7.792), had a negative effect on seasonal home range size. For coyotes, MDEV (ß_MDEV_ = -1.413) and NATR (ß_NATR_ = -11.453) had negative effects on home range size, while OPEN (ß_OPEN_ = 0.753) had a positive effect. For red foxes, MDEV (ß_SPEC:MDEV_ = 5.616) and NATR (ß_SPEC:NATR_ = 18.257) both had positive effects on home range size, while OPEN (ß_SPEC:OPEN_ = -5.719) had a negative effect.

**Table 6:** Results of *a-priori* model comparisons for selecting factors driving seasonal home range size in urban coyotes and red foxes in Madison, WI, 2015–2016.

Name	AICc	ΔAICc	AIC_w_	Cumulative AIC_w_
Species-specific use of NATR and MDEV	188.59	0.00	0.60	0.60
Species-specific use of NATR and OPEN	189.44	0.85	0.39	0.99
Species-specific use of NATR	196.65	8.06	0.01	1.00
Species-specific use of HDEV	203.01	14.42	0.00	1.00
Species-specific use of OPEN	204.55	15.95	0.00	1.00
Species-specific use of MDEV	205.17	16.58	0.00	1.00
Species, Sex, and Season	206.66	18.07	0.00	1.00
Species and Season	209.40	20.80	0.00	1.00
Season	210.23	21.63	0.00	1.00
Species and Sex	210.36	21.77	0.00	1.00
Sex	211.35	22.75	0.00	1.00
Species	214.13	25.54	0.00	1.00

### Annual habitat composition

**Fig 3:**
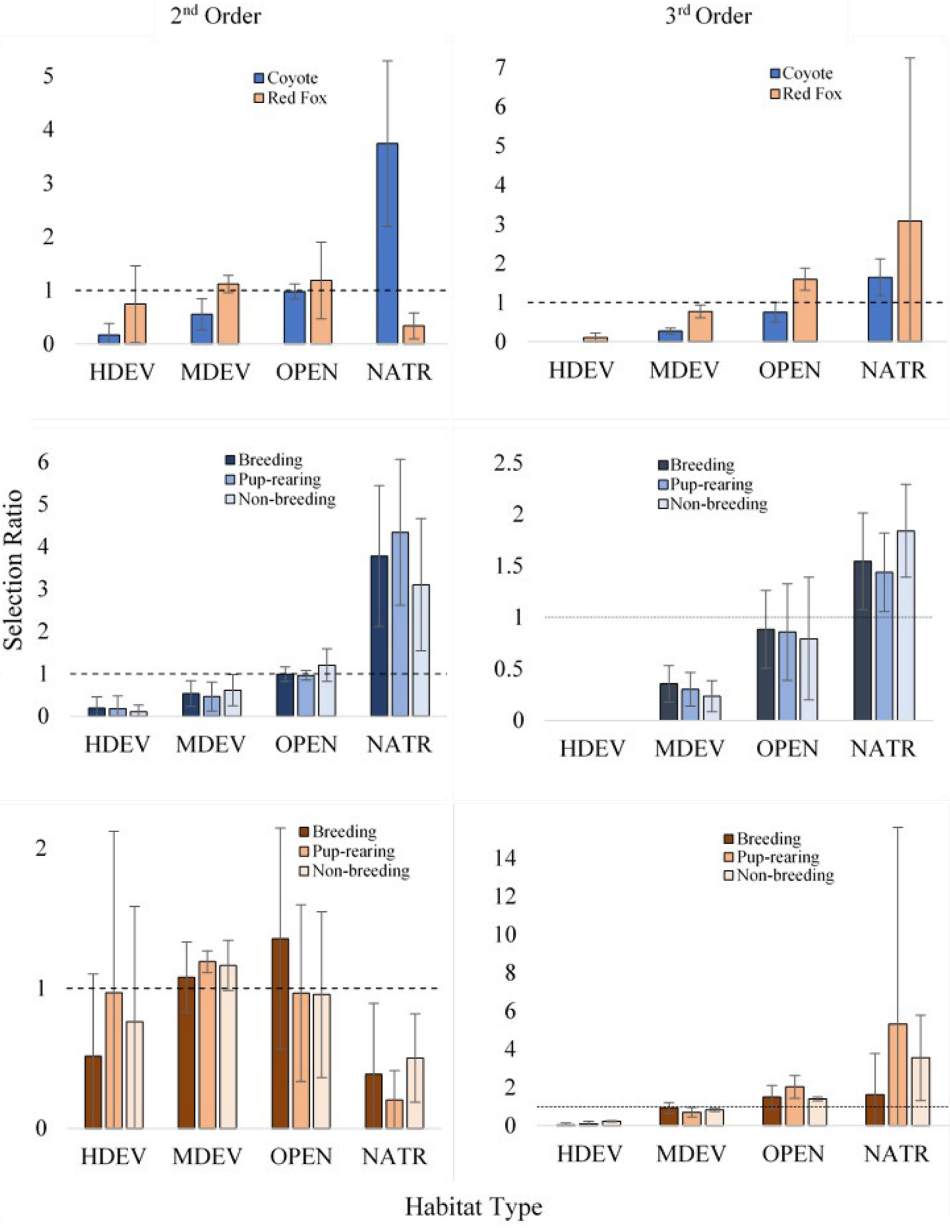
Habitat selection of coyotes (rows 1 and 2) and red foxes (rows 1 and 3) in Madison, WI, 2015–2016. 2^nd^ order (left) indicates home range selection compared to available habitat, 3^rd^ order (right) indicates use of actual locations compared to available habitat within an individual’s home range. Selection ratios (percent use/percent available) greater than 1 indicate selection, less than 1 indicate avoidance.

At the 2^nd^ order scale, both coyotes (p = 0.003) and red foxes (p < 0.001) differentially selected for habitat within our study area. Coyotes selected home ranges with NATR and avoided HDEV and MDEV. Red foxes used HDEV and MDEV in proportion to their availability and avoided NATR (Fig 3).

At the 3^rd^ order scale, both coyotes (p < 0.001) and red foxes (p = 0.002) differentially selected for habitat within their respective home ranges. Coyotes selected for NATR, avoided MDEV, and used OPEN in proportion to availability. Red foxes avoided MDEV, selected for OPEN, and used NATR in proportion to its availability (Fig 3).

### Seasonal habitat composition

Coyote selection patterns did not vary across seasons. At the 2^nd^ order scale, coyotes selected home ranges with NATR, avoided HDEV and MDEV and used OPEN in proportion to its availability on the landscape (Fig 3). At the 3^rd^ order scale, coyotes again selected for NATR, avoided MDEV, and used OPEN in proportion to its availability within the home range (Fig 3).

During the breeding season, at the 2^nd^ order scale, red foxes avoided NATR and used HDEV and MDEV, as well as OPEN in proportion to its availability on the landscape (Fig 3). At the 3^rd^ order scale, foxes used all available habitat in proportion to its availability (Fig 3). During the pup-rearing season, at the 2^nd^ order scale, red foxes exhibited selection for MDEV, avoided NATR and used HDEV and OPEN in proportion to its availability on the landscape. At the 3^rd^ order scale, foxes avoided HDEV and MDEV, selected for OPEN and used NATR in proportion to its availability in the home range. During the non-breeding season, at the 2^nd^ order scale, foxes avoided HDEV and NATR, but used other habitats in proportion to their availability on the landscape. At the 3^rd^ order scale, foxes selected for NATR and OPEN, and avoided HDEV and MDEV.

### Overlap of habitat use

Based on Pianka’s index, overlap of coyote and red fox habitat use differed at both the home range (0.68) and location level (0.41). Overlap of habitats also differed seasonally. At the home range level, habitat use overlap was less during the pup-rearing season (breeding = 0.70, pup-rearing = 0.55, non-breeding = 0.76). At the location level, habitat use overlap was low, but was relatively greater during the pup-rearing season (breeding = 0.32, pup-rearing = 0.42, non-breeding = 0.39).

### Movement/Step length

**Fig 4:**
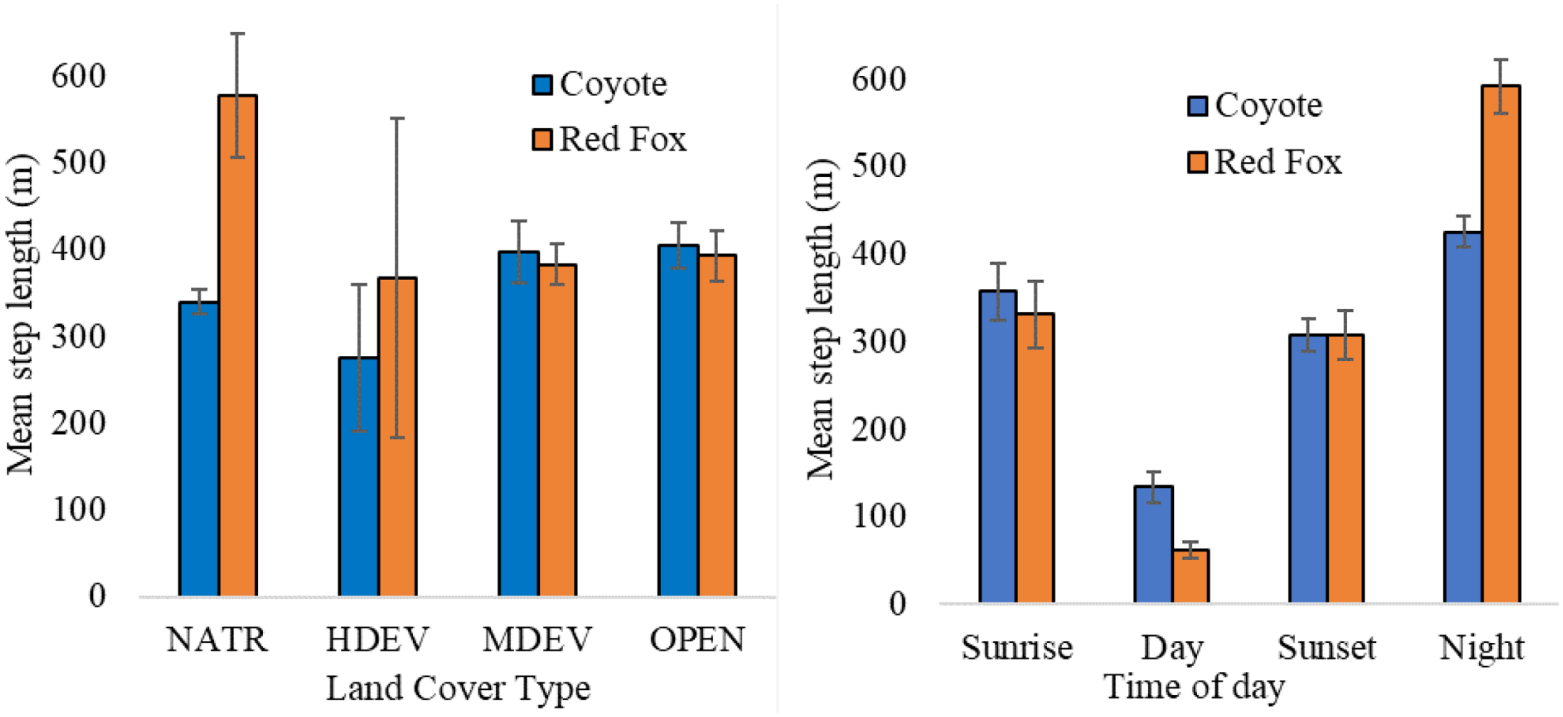
Mean step length (distance between two subsequent locations collected one-hour apart) for coyotes and red foxes by habitat type (left) and time of day (right) in Madison, WI, 2015–2016.

Habitat type had a significant effect on step length for both coyotes (F_4, 1434_ = 4.151, p = 0.002) and red foxes (F_4, 806_ = 4.176, p = 0.002) (Fig 4). Post-hoc Tukey HSD tests showed that for foxes, steps originating in NATR ($\bar{x}=577.59\text{m}$) were significantly longer than steps originating in OPEN ($\bar{x}=393.42\text{m}$, p = 0.025) and MDEV ($\bar{x}=383.46$, p < 0.001). For coyotes, steps originating in NATR ($\bar{x}=340.18\text{m}$) were significantly shorter than steps originating in OPEN ($\bar{x}=405.16\text{m}$, p = 0.005). Step length did not significantly differ for steps originating in the remaining habitats for either species.

Time of day significantly affected step length for both coyotes (F_3, 1435_ = 45.600, p < 0.001) and red foxes (F_3, 807_ = 50.380, p < 0.001) (Fig 4). For both species, steps recorded during night periods (${\bar{x}}_{\text{fox}}=591.72\text{m}$; ${\bar{x}}_{\text{coyote}}=425.05\text{m}$) were significantly longer than steps recorded during day (${\bar{x}}_{\text{fox}}=62.14\text{m}$, p_fox_ < 0.001; ${\bar{x}}_{\text{coyote}}=134.10\text{m}$ p_coyote_ < 0.001), sunrise (${\bar{x}}_{\text{fox}}=331.15\text{m}$, p_fox_ < 0.001; ${\bar{x}}_{\text{coyote}}=357.07\text{m}$ p_coyote_ < 0.001), and sunset periods (${\bar{x}}_{\text{fox}}=307.72\text{m}$, p_fox_ < 0.001; ${\bar{x}}_{\text{coyote}}=307.58\text{m}$, p_coyote_ < 0.001). Steps collected during crepuscular time periods (sunrise, sunset) were also significantly longer than steps in day periods (p_fox_ < 0.001, p_coyote_ < 0.001).

Sex significantly affected step length for coyotes (F_1, 1437_ = 9.106, p = 0.003) and red foxes (F_1, 809_ = 63.09, p < 0.001). For coyotes, females ($\bar{x}=390.07\text{m}$) displayed significantly longer steps than males ($\bar{x}=346.49\text{m}$, p = 0.003). For red foxes, males ($\bar{x}=508.66\text{m}$) displayed significantly longer steps than females ($\bar{x}=272.77\text{m}$, p < 0.001).

Season significantly affected step length for red foxes (F_2, 808_ = 11.93, p < 0.001), but not for coyotes (F_2, 1436_ = 0.414, p = 0.661). Post-hoc Tukey HSD tests showed that fox steps during the pup-rearing season ($\bar{x}=315.75\text{m}$) were significantly shorter than those during the breeding ($\bar{x}=493.00\text{m}$, p = 0.007) or non-breeding season ($\bar{x}=439.21\text{m}$, p < 0.001).

For red foxes, proximity to coyote core use area did not significantly alter step length (F_2, 836_ = 0.886, p = 0.41) or step direction (*X*^2^_2_ = 4.495, p = 0.087).

## Discussion

Our research shows how coyotes and red foxes partition space resources within an urban landscape. The spatiotemporal use of our urban study area by coyotes and red foxes appear dictated by habitat type within the human-dominated landscape. Like rural canids [19, 42], it appears that coyotes and red foxes both displayed intraspecific territoriality within our urban study area, but there may be a greater degree of interspecific overlap between coyotes and foxes in urban areas relative to rural ones [25,55]. Coyotes selected for relatively large NATR in their territories, and avoided areas of HDEV; while foxes avoided NATR and instead primarily selected for OPEN within their home ranges. This species-specific habitat use also appeared to be the primary driver of both annual and seasonal home range sizes of coyotes and red foxes. While their movement patterns differed based on species-specific habitat use, coyotes and red foxes were both active at similar times of the day.

Across temporal and spatial scales, coyotes selected for NATR, and avoided MDEV and HDEV. This pattern suggests that natural areas are important for coyotes inhabiting urban environments, and despite living in close proximity to humans in the city, coyotes generally avoided them [49]. MDEV and HDEV potentially have more human-use when compared to NATR or OPEN [40], and studies have shown that vehicles are a primary source of mortality for urban coyotes [50]. These factors could explain why coyotes consistently avoided human-dominated areas. Urban coyotes typically maintain a largely natural diet [51], and may be able to satisfy their dietary requirements primarily in natural areas, where they can avoid humans and the potential risks associated with MDEV and HDEV despite urban areas being a rich source of anthropogenic food.

In many rural areas coyotes appear to drive the distribution of sympatric red foxes through spatial exclusion [25, 27–28]. Our results show the same dynamic may occur in urban areas but to a lesser degree. Coyotes and red foxes displayed similar habitats within their home ranges, but the amounts of habitat types within home ranges differed by species. While coyotes selected home ranges with large NATR components, red foxes avoided these areas and used other areas in proportion to their availability on the landscape. Red foxes used most areas in proportion to their availability, as would be expected from a generalist [22, 42]. While coyotes are also generalists, they act as apex carnivores in urban areas [30] and may select for their preferred habitat. Foxes largely avoided areas that were preferred by coyotes, even though foxes frequently used similar areas within their home ranges, suggesting that on the landscape level, a degree of interspecific spatial partitioning may be occurring.

Species-specific habitat use appeared to be the most important factor affecting annual home range size for both coyotes and red foxes. Our top model—species-specific use of HDEV—showed that coyotes and foxes responded differently to the amount of HDEV within their annual home range. Resource availability often dictates home range size and home ranges for synanthropic species are generally smaller than for their rural counterparts due to increased and more concentrated resource availability [6, 17]. We found that as the proportion of HDEV within a coyote’s home range increased, home range size would also increase, suggesting that coyotes may rely on other habitats to satisfy their ecological needs. Red fox home ranges were not negatively affected by development in the way that coyotes were, and despite avoiding HDEV it made up a larger proportion of their home ranges.

Species-specific habitat use within the home range best explained the variation in seasonal home range size as well, although limited samples may have affected the accuracy of our results. Coyotes primarily selected for NATR, likely to satisfy resource requirements [50], so higher proportions of this habitat type may allow coyotes to successfully acquire resources without needing to cover and defend larger expanses of territory [6]. We predicted the opposite trend for red foxes; when the proportion of NATR increased, home range size would increase. On the landscape level (3^rd^ order), red foxes avoided NATR, so it may be expected that an increase in the proportion of NATR within a red fox home range would cause an increase in home range size to successfully acquire adequate resources [52–53]. There was less overlap in home range composition during the pup-rearing season compared to the breeding and non-breeding seasons, suggesting that at the home range level, red foxes and coyotes are more spatially segregated during the time young are born and being cared for, a behavior that has been observed in sympatric rural populations of coyotes and red foxes [27–28].

Habitat and development also affected the fine-scale movements of urban canids, with both canid steps being longer in habitats that were selected neither for nor against. Longer step lengths by coyotes in HDEV may be because they quickly passed through these areas, while longer steps by foxes in NATR may be to escape detection by coyotes. Despite known exclusion by coyotes in rural areas [25–26], fox step length and direction did not significantly change when closer to coyote core-use areas. This may be attributed to the ambiguity of a 50% core use or 95% MCP boundary, or that red foxes are indifferent to coyote presence in habitats that both species frequent, or other factors, such as prey distribution. Both coyotes and red foxes displayed temporal variation in step length, generally being more active when humans are less active. This trend is well supported for urban coyotes [50], but to our knowledge, this is the first documentation for urban red foxes. The variation in step length and habitat selection may help to facilitate coexistence of canids in urban areas by allowing the spatiotemporal partitioning of resources [8].

Season and an individual’s sex also affected step length. Female coyotes and male red foxes displayed longer steps. We detected no seasonal difference in step length for coyotes, but significantly shorter fox steps during the pup-rearing season. During this season, both coyotes and red foxes use den sites to raise pups, however only a small proportion of a coyote pack frequently uses the den site [19], unlike red foxes where most individuals localize their movements and return to the den site daily [42]. Coyotes—as apex carnivore—may not feel predation pressure and would not need to be as strongly tied to the den site to protect pups [6, 30]. This may explain why we detected a difference in fox step length during this time of the year, as foxes seemed to be concentrated around this central den site once pups were born. We suspect that a high degree of individual variation in coyote activity could have masked a true difference in step length between seasons for our study, as coyotes generally have a wide range of individual social roles [19], but we did not account for the role of an individual within a pack or family in our study.

Coyotes and red foxes in Madison generally appeared to be spatially partitioning use of our urban study area, but displayed various degrees of overlap between interspecific home ranges. In rural contexts, spatial partitioning with some overlap is well documented [25, 27–28] and suggests that red foxes avoid areas used extensively by coyotes. A similar mechanism may exist to facilitate their coexistence in a spatially constrained urban environment. If coyotes in urban areas avoid areas used by humans and our research suggests that urban red foxes select for more developed areas, it could be hypothesized that red foxes may be using these developed areas as refugia to avoid coyotes [25]. We could not definitively describe the mechanism for this selection, specifically if red foxes were self-selecting for these areas or if they were using them as refuge from coyotes occupying natural areas. Studies of urban red foxes in Europe (an area absent of coyotes) suggested that density was proportional to the amount of moderate-density housing available [22] and that in a particular urban area that lacked coyotes, red foxes used residential and developed open areas disproportionately to their availability [25], suggesting that red foxes may not be selecting developed areas only to avoid coyotes, but for other reasons as well.

**Fig 5:**
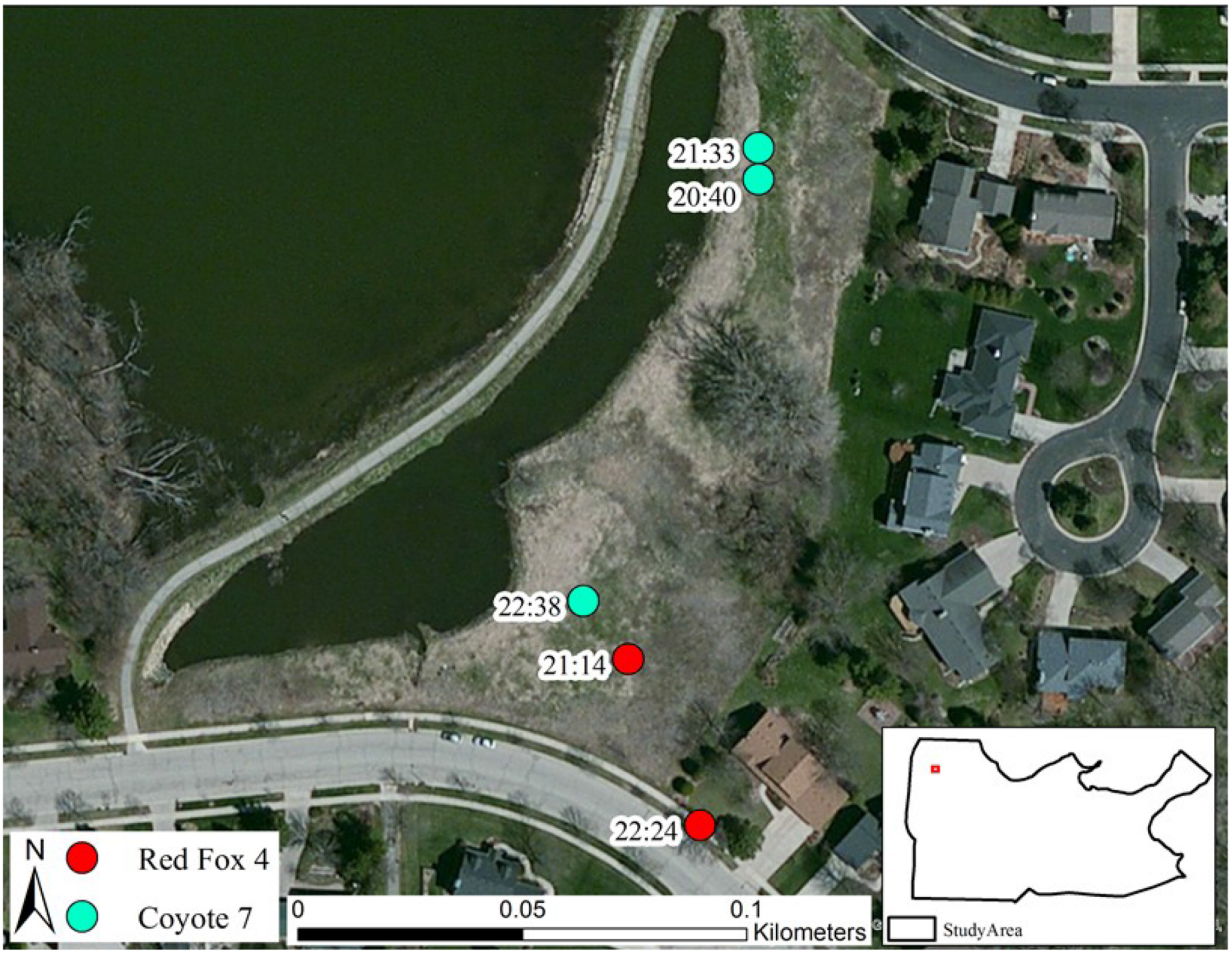
Radio-collared coyote and red fox sharing an urban field while hunting independently, with no aggressive interactions, Madison, WI, October 12, 2015.

**Fig 6:**
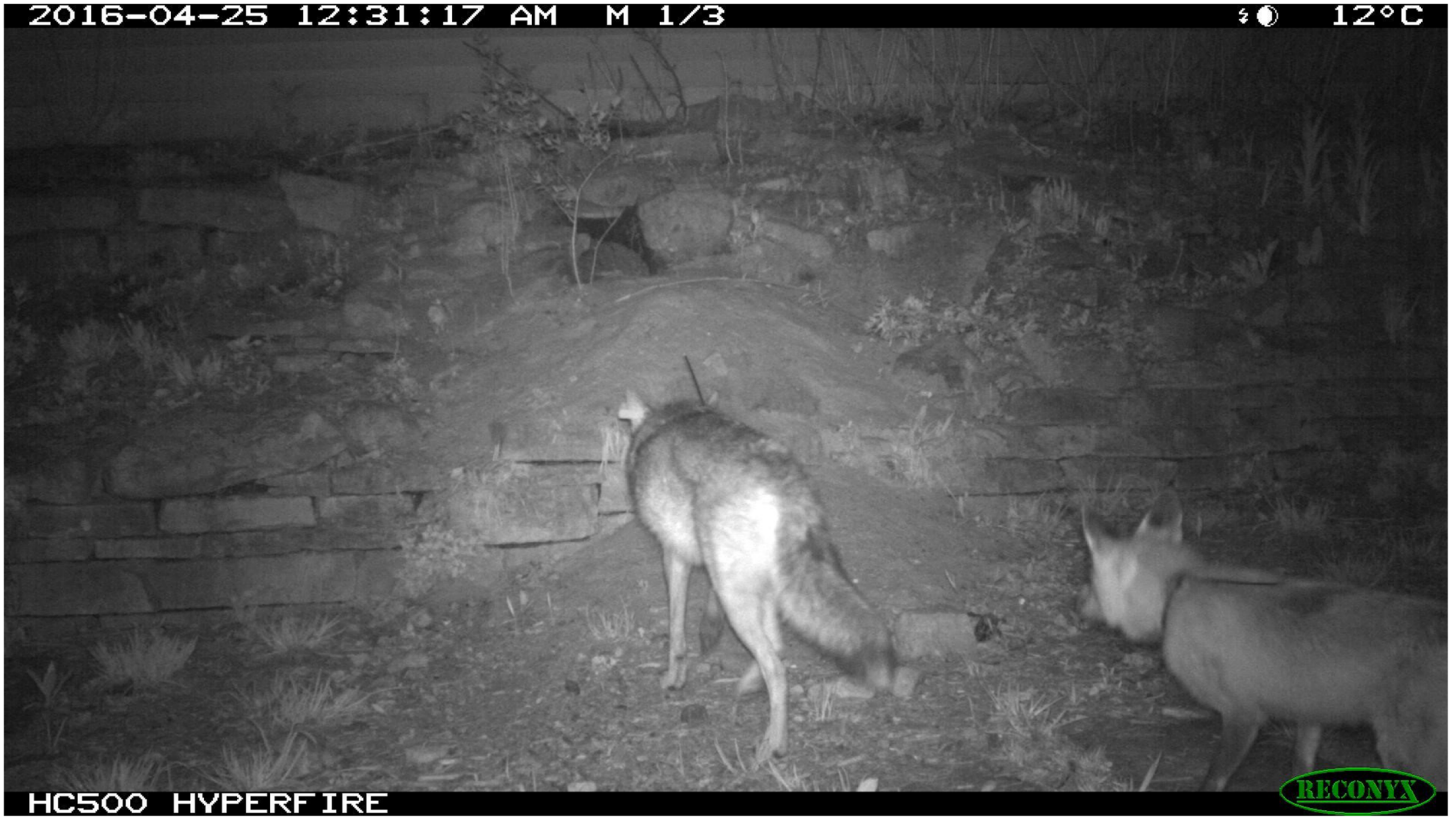
Coyote (left) investigating active red fox (right) den. Pair of coyotes visited den site weekly, even scavenging a cottontail rabbit carcass at one point. Behavior continued for close to a month and red foxes never relocated. Madison, WI, April 26, 2016.

Despite the evidence of habitat partitioning to facilitate coexistence between these two sympatric canid species, during the duration of our research several interspecific interactions between coyotes and red foxes provided evidence that there may be more to this relationship. For example, a radio-collared coyote and red fox were observed foraging < 100m from one another for over 1 hour in an open field with no sign of aggression or negative interactions (Fig 5). In another example, a pair of coyotes frequently visited (1–2 visits/week for 4 weeks) an active red fox den in a residential neighborhood (Fig 6). While the coyotes investigated the den site and partially entered the den to retrieve an Eastern cottontail (*Sylvilagus floridianus*) carcass, the adult foxes remained in the vicinity. This behavior continued for close to one month, with no attempts from the red fox family to relocate to any of several available nearby dens. Coyotes and red foxes may be more tolerant of each other in urban areas than we initially suspected. In either situation, if the red foxes perceived coyotes as dangerous, it would be expected that they would have altered their behavior to avoid a negative interaction [54–55].

While the focus of our study was not on food or habitat resource availability, we suspect that the mechanism that facilitates coexistence in urban areas is rooted in an abundance of food resources in our urban study area [1, 56]. More abundant resources appear to allow both species to display smaller home ranges, which may allow for these two traditionally competitive species to coexist within urban environments with a similar dynamic to rural coyotes and red foxes, but on a smaller scale with potentially less competitive interactions [25–26]. Habitat patch size—especially of NATR—may also be important to home range size and composition. In our study area, coyotes within the largest natural area (UW Arboretum) rarely ventured into surrounding developed areas, whereas coyotes near smaller natural areas (Owen Park) frequently used adjacent neighborhoods. The size of natural areas may influence resource availability and therefore affect the way wildlife uses these areas. Additional research should be conducted using fine-scale tracking techniques and GPS technology on sympatric canids to investigate the potential for interactions between these two species to adequately determine if coyotes are displacing red foxes or if red foxes are simply self-selecting for more developed areas of urban landscapes.

## Supporting information

S1 File Occurrence data for red foxes and coyotes in Madison, WI, 2015–2016. (CSV)
